# Supplementary figures and images for: A minimalist computational model of slice hippocampal circuitry based on Neuronify for teaching neuroscience
Source: PLoS One. 2025 Apr 29;20(4):e0319641. doi: 10.1371/journal.pone.0319641 (PMC12040273; doi:10.1371/journal.pone.0319641)

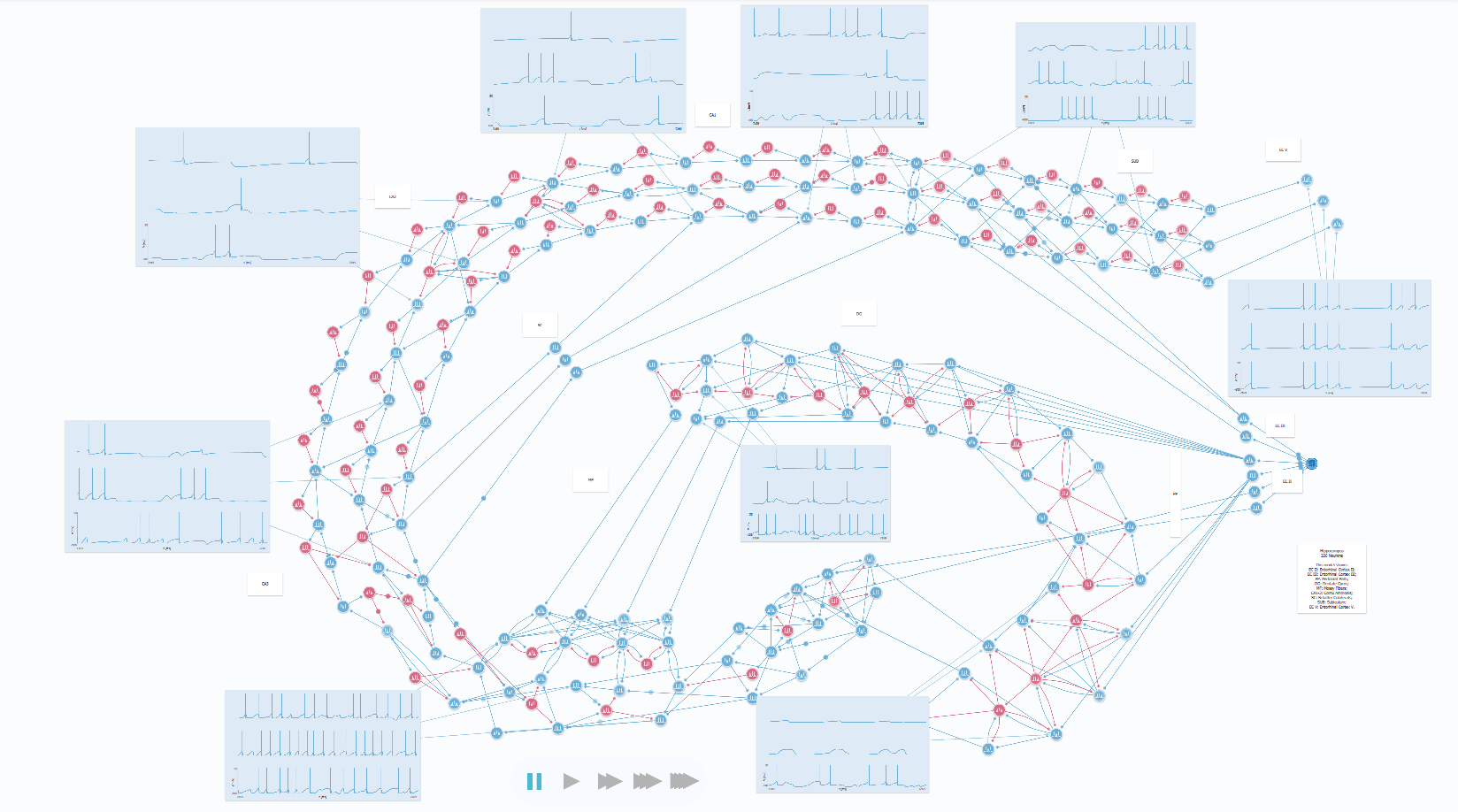

Supplement: S1 Fig — Wide view of the educational model working with an irregular spike generator stimulating the EC II and EC III neurons. Altough most of the other spike patterns are similar to Fig 5, note the presence of apparent bursts in DG and in the CA3-CA2 transition. (TIF) [file pone.0319641.s001.tif]
