# Supplementary material for: A minimalist computational model of slice hippocampal circuitry based on Neuronify for teaching neuroscience
Source: PLoS One. 2025 Apr 29;20(4):e0319641. doi: 10.1371/journal.pone.0319641 (PMC12040273; doi:10.1371/journal.pone.0319641)
Supplement: Attachment — Submitted filename: Response_to_Reviewers_auresp_2.pdf [file pone.0319641.s004.pdf]

Response to Reviewers

Reviewer #1: *The revised manuscript is much improved. I thank the authors for their thoughtful revisions. I believe the manuscript is now ready for publication, except for a few minor clarifications, listed below.*

R: The authors thank the reviewer for the consideration and for the meaningful and constructive comments. The Manuscript was revised considering reviewer’s comments and changes are trackable in the “track changes” version of the Manuscript. Additionally, at the conclusion of this response, we have compiled a list of the references that were revised in the first revision to achieve the journal requirements.

1) *The text refers to "Cornus Ammonis". The correct term is "Cornu Ammonis".*

R: We appreciate the correction regarding the term “Cornu Ammonis”. We’ve updated the text accordingly to reflect this accurate terminology.

2) *p. 6, the text "fourth, the information cadenced flow with collateral excitation of CA1; fifth, the relative temporal synchronization of spikes as output through simultaneous excitation coupled to inhibitory feedback of SUB". The meaning of this is really unclear, particularly because of the use of non-standard terminology such as the "information cadenced flow". Please revise this so that the meaning is clear to neuroscientists.*

R: We thank the reviewer for the important comment on our lexical choices, we seeked the improvement of our writing on this excerpt by adapting the highlighted expressions by others more adequate to the neuroscientific context. The novel composition of this paragraph follows:

“[...] fourth, the action potentials are feedforwardly transmitted with collateral excitation of CA1; fifth, the almost simultaneous spikes' generation between layers as output of SUB, achieved through simultaneous excitation coupled to inhibitory feedback.”

3) *The order of figures 3,4,5 and the corresponding text describing the models are quite confusing. Consider starting with the current text on microcircuit motifs, then describe the "complete" HP circuit model, then describe the reduced and educational models. Or consider alternative narrative sequences that are more logical than the current text.*

R: We agree that the flow of the description could be improved for better clarity. Based on your suggestion, we have revised the manuscript to enhance the narrative structure. We have now started the materials and methods section by introducing the software (Neuronify), followed by an explanation of the methodology used (motif-based approach and model building). We then describe the models in a new subsection “Hippocampal circuitry models” which begins with the educational (or complete) version and ends explaining the simplified model. Finally, we described the model validation. Also, the educational version of the HP model was referred in the text as both “educational” and “complete”, to avoid confusion, we changed all mentions of “complete” to “educational”. We believe these changes have significantly improved the overall narrative clarity and flow.

4) *Figure 4 appears to be truncated in the current manuscript, and it is difficult to understand how it fits in the narrative. Please correct it so that it fits on the page, and consider moving it after the complete model diagram.*

R: We appreciate your insightful comment. Upon further consideration, we agree that the inclusion of that figure in its current position does not fit the narrative as it shows a small portion of the simplified model before the whole model is introduced in Figure 5, which is an order that is very likely to cause confusion. In response, we have decided to remove the figure for greater clarity as it is also redudant since the spike patterns it intends to show appear later in the figures 13 and 12.
(The figures 5, 13, and 12 correspond to the figures 4, 6, and 7 in the new version of the manuscript.)

5) *Figure 5: Panels b) and d) need labels so that they can be related to c) and e). Consider moving panel c) after panel d), as it is conceptually closest to the model.*

R: Thank you for your insightful feedback about our image. We made the required modifications in figure 5.

6) *p. 8 "The input patterns are generated by a continuous current that simulates the stimulus's arrival from the EC region. In this context, we prioritized standardizing the input ... one of our goals is to show a constant frequency of stimuli flowing downstream through the Hippocampus as its subregions modify it with their specific..."*

- I do not fully understand the meaning of this. Is the intent to have constant input through sustained current injection in order to be able to better observe the transformations of the input through the different HP regions? In that case, please clarify this paragraph and consider adding a supplementary figure that shows what happens when irregular input is

provided to the model, as a way to show that more realistic inputs are possible in the model framework.

Yes, the intent is indeed to have a constant input so the effect of motifs on the spike patterns can be observed better. We rewrote the paragraph in order to clarify it and also moved that paragraph so it appears before the model description begins. We also added a supporting figure (S1) showing the effect of a random spike generator stimulating the model, aiming to exemplify the importance of constant stimulation for maintaining the characteristics expected by the model as a representation of hippocampal functioning.

7) *Please clarify in the text what is the model in Figure 5: is this the complete model? Table 1 does not show the numbers for the complete model in Fig. 5. Please modify it so that it mirrors the corresponding narrative in the text.*

R: We appreciate your comment. In the text, we described the completed version as an “educational model”, including in the table, showing the numbers of neurons (excitatory and inhibitory) in each region of the hippocampus simulating the human brain. In response to the comment, we modified the figure capture to make it clearer, and to standardize, we chose to refer to it as the “educational model” in all instances, including in the abstract.

8) *Fig 6 - Fig 11: consider replacing only with Fig. 11, otherwise there are too many figures in the manuscript and it is difficult to follow, especially with the low resolution images. There is redundancy with Fig. 13. Instead of Fig. 6 - Fig. 14, consider including only the current Fig. 11 (complete model), Fig. 12 (reduced model), Fig. 13 (comparison between models), Fig. 14 (CA3 backprojection).*

R: We appreciate your insightful feedback. In response we deleted figure 6 to figure 10 to reduce the number of figures in the manuscript.

9) *Fig. 14 - due to the low image resolution, I am unable to see which neurons have their spiking activity plotted. Does Figure 14B refer to the altered activity of DG granule cells due to the added CA3c backprojection? Please clarify in the figure caption.*

R: Thank you for your valuable feedback. We apologize for the low image resolution in Figure 14, which may have made it difficult to properly analyze its content. In response we

improved the quality of the figure. The figure 14B shows a voltmeter, which measures the spikes of modified CA3c neurons. We intended to show how the motifs influence the spiking activity, once reducing recurrent excitation and adding inhibitory motifs, the desired CA3c burst pattern is created. We modified the figure capture for clarification.

Journal requirements*: Please review your reference list to ensure that it is complete and correct. If you have cited papers that have been retracted, please include the rationale for doing so in the manuscript text, or remove these references and replace them with relevant current references. Any changes to the reference list should be mentioned in the rebuttal letter that accompanies your revised manuscript. If you need to cite a retracted article, indicate the article's retracted status in the References list and also include a citation and full reference for the retraction notice.*

R: To address this request, we carefully revised the reference list and compared the original manuscript with the revised version. In the revised version we used LaTeX to The following modifications were made:

New references added:

[17] *Burkitt AN. A Review of the Integrate-and-fire Neuron Model: I. Homogeneous Synaptic Input. Biological Cybernetics. 2006;95(1):1–19. doi:10.1007/s00422-006-0068-6* (included in Neuronify subsection in Materials and Methods)

[22] Amaral DG, Witter MP. The three-dimensional organization of the hippocampal formation: A review of anatomical data. Neuroscience. 1989;31(3):571–591. doi:10.1016/0306-4522(89)90424-7.

[27] *Stewart M. Antidromic and orthodromic responses by subicular neurons in rat brain slices. Brain Research. 1997;769(1):71–85. doi:10.1016/S0006-8993(97)00690-2.* (included in Simplified HP circuitry model subsection in Materials and Methods)

[52] *Scharfman HE. The CA3 "backprojection" to the dentate gyrus. In: Progress in Brain Research. vol. 163. Elsevier; 2007. p. 627–637. Available from: https://linkinghub.elsevier.com/retrieve/pii/S0079612307630349* (included in Model Variants subsection in Discussion)

[25] *Sik A, Penttonen M, Ylinen A, Buzsaki G. Hippocampal CA1 interneurons: an in vivo intracellular labeling study. The Journal of Neuroscience. 1995;15(10):6651–6665. doi:10.1523/JNEUROSCI.15-10-06651.1995.* (included in Simplified HP circuitry model subsection in Materials and Methods)

References excluded:

-Li, Y.; Mu, Y.; Gage, F. H. Development of Neural Circuits in Adult Hippocampus. Current Topics in Developmental Biology, v. 87, p. 149–174, 2009.

- Giap et al. The Hippocampus Anatomy, Pathophysiology, and Regenerative Capacity. Journal of Head Trauma Rehabilitation, v. 15, n. 3, p. 875–894, 2000.

- Gergues. M. et al. Circuit and molecular architecture of a ventral hippocampal network. Nature Neuroscience, v. 23, p. 1444–1452, 14 set. 2020.

- Latimer, B. et al. Open Source Software Tools for Teaching Neuroscience. The Journal of Undergraduate Neuroscience Education, v. 16, n. 3, p. A197–A202, 3 set. 2018
